# Supplementary material for: Unveiling the bosonic nature of an ultrashort few-electron pulse
Source: Nat Commun. 2018 Jul 18;9:2811. doi: 10.1038/s41467-018-05203-7 (PMC6052057; doi:10.1038/s41467-018-05203-7)
Supplement: Supplementary file 1 — Supplementary Information [file 41467_2018_5203_MOESM1_ESM.pdf]

Supplementary Information: Unveiling the bosonic nature of an ultrashort few-electron pulse

Gregoire Roussely†,[1] Everton Arrighi†,[1] Giorgos Georgiou,[1,2] Shintaro Takada,[1,3] Martin Schalk,[1] Matias Urdampilleta,[1] Arne Ludwig,[4] Andreas D. Wieck,[4] Pacome Armagnat,[5] Thomas Kloss,[5] Xavier Waintal,[5] Tristan Meunier,[1] and Christopher Bäuerle[1]

[1)] *Univ. Grenoble Alpes, CNRS, Grenoble INP, Institut Néel, 38000 Grenoble, France*

[2)] *Univ. Savoie Mont-Blanc, CNRS, IMEP-LAHC, 73370 Le Bourget du Lac, France*

[3)] *National Institute of Advanced Industrial Science and Technology (AIST), National Metrology Institute of Japan (NMIJ), Tsukuba, Ibaraki 305-8563, Japan*

[4)] *Lehrstuhl für Angewandte Festkörperphysik, Ruhr-Universität Bochum, Universitätsstrasse 150, 44780 Bochum, Germany.*

[5)] *Univ. Grenoble Alpes, CEA, INAC-Pheliqs, 38000 Grenoble, France*

SUPPLEMENTARY NOTE 1

Number of injected charges

The number of electrons injected into the quasi-1D channel can be arbitrarily tuned by the voltage applied on the left ohmic contact, V_{p} [1],

$$n_e = 2\frac{e}{h}\int V_{\mathrm{p}}(t)\ dt,$$

where n_e is the number of excited electrons, h is Plank's constant and e is the elementary charge. In this formula we assume a single channel of conductance as well as spin degeneracy. The voltage on the left ohmic contact, $V_{\mathrm{p}}(t)$, can be calculated directly from the voltage amplitude applied by the AWG on the RF line and by taking into account the appropriate line attenuation.

To estimate the number of generated electrons contained in one electron pulse we measure the rectified current across a 10 kΩ resistor, which is amplified and measured with a lock-in amplifier. The measured signal is then proportional to the number of generated electrons $\overline{n}_e$ distributed over all available conduction channels and to the repetition rate of our AWG, which is $f = 600$ MHz, i.e.,

$$I = \overline{n}_e\ e\ f = \frac{V_{\mathrm{rms}}}{g}\ \frac{1}{10\ \mathrm{k}\Omega}\ \frac{\pi}{\sqrt{2}}$$

where V_{rms} is the voltage measured with the lock-in amplifier, $g = 1000$ is the amplifier gain and the constant factor $\pi/\sqrt{2}$ is to account for the 12 kHz square pulse modulation signal used for the lock-in detection. As shown in Fig. 1d of the manuscript we can excite from 0.6 to 6.1 electrons per pulse by varying the amplitude of the input pulse.

SUPPLEMENTARY NOTE 2

Time calibration of RF lines

The RF lines used for the excitation and detection of the electron pulse are calibrated using two methods, reflectometry measurements and in-situ calibration.

To perform the reflectometry calibration we initially send a short pulse through the RF line and measure the reflected pulse with a high bandwidth oscilloscope. As shown in Supplementary Figure 1 we can estimate the relative time delay between the four RF lines by looking at the reflected pulses. This approach offers limited time accuracy ($\approx \pm$ 5 ps) since the RF lines cannot be calibrated with the attenuators installed. A second and more precise calibration can be done in-situ by exploiting the fast propagation of the 2D plasmon.

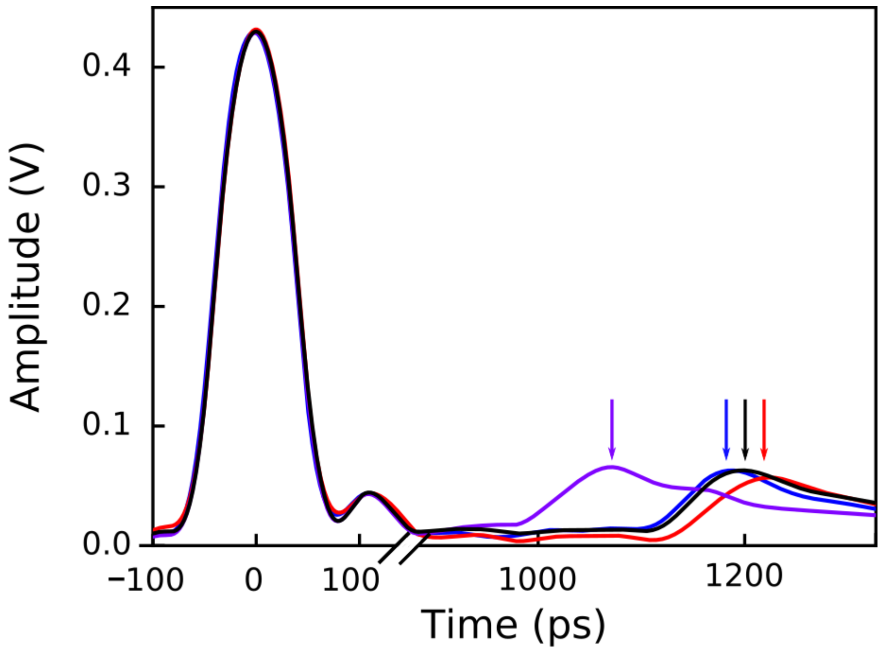

Supplementary Figure 1. **Time domain reflectometry**. The large amplitude curves at 0 ps are the pulses applied on the RF lines, while the smaller amplitude curves above 1000 ps are the reflected pulses. The four arrows indicate the peak of the reflected pulse connected to the ohmic contact (blue curve), QPC_1 (red curve), QPC_2 (purple curve), QPC_3 (black curve).

To do so, we almost completely depolarise the one-dimensional channel gates. The collective motion of electrons in this almost confinement-free system has the velocity of a

two-dimensional plasmon, which is well known to be $v^{2D}_{plasmon} \sim 10^7\,\mathrm{m\,s^{-1}}$ [2,3]. When the two-dimensional plasmon is injected at the left ohmic contact it will reach the sampling QPC essentially instantaneously. By measuring a time resolved trace of the two-dimensional plasmon pulse we can estimate the relative time delay between the excitation RF line and the detection QPC RF lines. This allows us to calibrate our RF lines at low temperatures with an accuracy in the order of 2 ps.

SUPPLEMENTARY NOTE 3

Time-of-flight measurements – Velocity calculation

The velocities shown in Fig. 2b of the manuscript (blue data points) are derived from time-of-flight measurements. As it was briefly explained in the methods section, to determine the velocities we performed three independent measurements, one for each QPC (except QPC_0 which is not connected to a bias tee). For each measurement we monitored the time of flight of the electron wavepacket from the left ohmic contact to the respective QPC, resulting into t_1, t_2 and t_3 for QPC_1, QPC_2 and QPC_3 respectively. The distance of the three QPCs from the left ohmic contact are derived from the Scanning Electron Microscope (SEM) image of our sample, shown in Fig. 4, and they are $d_1 = 15\,\mu m$, $d_2 = 30\,\mu m$ and $d_3 = 70\,\mu m$.

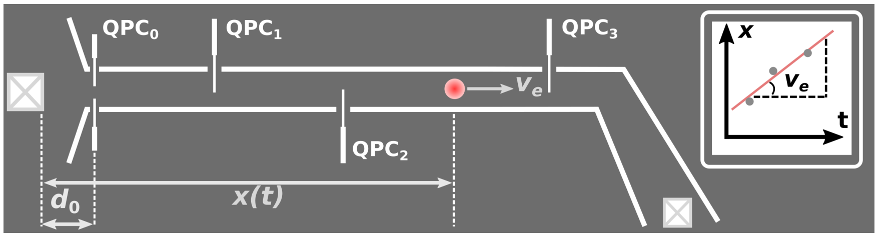

Supplementary Figure 2. **Velocity calculation**. Schematic illustrating the velocity calculation from the time-of-flight measurements. The electron wave packet (red sphere) is excited at the left ohmic contact (white crossed square) and it travels a distance d_0 before passing through the mode selection QPC_0 and entering the quasi one-dimensional wire. The wave packet will then travel at a speed v_e towards the three detection QPCs where a time-of-flight measurement is performed. The inset shows how the speed is derived from the three independent measurements.

As illustrated by Supplementary Figure 2, the electron wave packet excited at the left ohmic contact will cover a distance d_0 with an average speed v_0 before it reaches the quasi-1D channel. It will then travel towards the three QPCs with speed v_e. The equation governing the motion of the electron has the following form,

$$x = v_0 t_0 + v_e(t - t_0) = d_0 + v_e t \tag{1}$$

where x is the distance from the left ohmic contact to the three QPCs, t_0 is the time until the

electron wave packet reaches the quasi-1D channel and t is the time of flight. The velocity can be calculated from the slope of Supplementary Eq. (1) through a linear fit, as indicated by the inset of Supplementary Figure 2. The error bars on figure 2b of the main manuscript correspond to the velocity uncertainty obtained by the linear fit.

SUPPLEMENTARY NOTE 4

Simulating the plasmon dispersion relation from the microscopic model

A sketch of the system used in our simulations is shown in Figure 1c of the main text. We consider a 3D system with translational invariance along the x direction and with the 2D electron gas situated at $z = 0$. The two top gates, situated at $z = 140\,\mathrm{nm}$ are used for defining the quasi-one dimensional wire, but also provide screening to the electron gas.

Our starting point is a many-body Hamiltonian that describes our 2D electron gas,

$$H = -\frac{\hbar^2}{2m^*}\sum_\sigma \int d^2\mathbf{r}\; c^\dagger_{\mathbf{r}\sigma}\Delta c_{\mathbf{r}\sigma} + \sum_\sigma \int d^2\mathbf{r}\; U(\mathbf{r}) c^\dagger_{\mathbf{r}\sigma} c_{\mathbf{r}\sigma} + \sum_{\sigma\sigma'} \int d^2\mathbf{r} d^2\mathbf{r}' c^\dagger_{\mathbf{r}\sigma} c_{\mathbf{r}\sigma} G(\mathbf{r},\mathbf{r}') c^\dagger_{\mathbf{r}'\sigma'} c_{\mathbf{r}'\sigma'} \tag{2}$$

where the fermionic operator $c^\dagger_{\mathbf{r}\sigma}$ ($c_{\mathbf{r}\sigma}$) creates (destroys) an electron at position $\mathbf{r} = (x, y)$ and with spin σ, m^* is the effective mass, Δ the 2D Laplacian operator, $U(\mathbf{r})$ an electrostatic potential and $G(\mathbf{r},\mathbf{r}')$ the electron-electron interaction. In free space, $G(\mathbf{r},\mathbf{r}')$ is simply given by the bare Coulomb repulsion $G(\mathbf{r},\mathbf{r}') = \frac{e^2}{4\pi\epsilon|\mathbf{r}-\mathbf{r}'|}$. However, here, the presence of the electrostatic gates provides some screening, and $G(\mathbf{r},\mathbf{r}')$ is the solution of the 3D Poisson equation (restricted to the 2D gas),

$$\Delta_{3D} G(\hat{\mathbf{r}},\hat{\mathbf{r}}') = -\frac{e^2}{\epsilon}\delta(\hat{\mathbf{r}}-\hat{\mathbf{r}}') \tag{3}$$

with the boundary condition $G(\hat{\mathbf{r}},\hat{\mathbf{r}}') = 0$ when the 3D vector $\hat{\mathbf{r}} = (x, y, z)$ coincides with the position of a gate. $e > 0$ is the electron charge and ϵ the dielectric constant.

Self-consistent electrostatic-quantum problem

The first step in our calculation is a mean field (self-consistent Hartree) treatment of Supplementary Eq. (2), in which we aim at solving together the following equations,

$$\Delta_{3D} U(\hat{\mathbf{r}}) = -\frac{e\rho(\hat{\mathbf{r}})}{\epsilon} + \frac{e\rho_0(\hat{\mathbf{r}})}{\epsilon} \tag{4}$$

$$\frac{-\hbar^2}{2m^\star}\Delta\Psi(\mathbf{r}) - eU(\mathbf{r})\Psi(\mathbf{r}) = E\Psi(\mathbf{r}) \tag{5}$$

$$\rho(\mathbf{r}) = \sum_E f(E)|\Psi(E,\mathbf{r})|^2 \tag{6}$$

where $U(\mathbf{r})$ is the restriction of $U(\hat{\mathbf{r}})$ to the 2D plane of the electron gas, $\rho(\hat{\mathbf{r}}) = \rho(\mathbf{r})\delta(\mathrm{z})$, Ψ the electronic wave function, f the Fermi function and the continuum sum in Supplementary Eq. (6) spans over all the eigenstates of the Schrödinger Supplementary Eq. (5). The density ρ_0 accounts for the layer of dopants present above the 2D gas; we use $U(\hat{\mathbf{r}}) = V_{\mathrm{SG}}$ in the top gate and Von Neumann boundary conditions otherwise. We have explicitly verified that the finite width of the 2D gas along z does not play a role in these calculations. Translational invariance along x implies that the Poisson equation can be solved in the 2D (y, z) plane while the wave-function is a plane wave, which can be separated into a transverse and a longitudinal component $\Psi(E, \mathbf{r}) = e^{ik_\alpha(E)x}\psi_\alpha(y)$. Performing an explicit integration along the longitudinal direction at zero temperature, we arrive at,

$$\Delta U(y, z) = -\frac{e\rho(y)}{\epsilon}\delta(z) + \frac{e\rho_0(y, z)}{\epsilon} \tag{7}$$

$$-\frac{\hbar^2}{2m^\star}\frac{\partial^2}{\partial y^2}\psi_\alpha(y) - eU(y, 0)\psi_\alpha(y) = E_\alpha\psi_\alpha(y) \tag{8}$$

$$\rho(y) = \frac{2}{\pi}\sqrt{\frac{2m^\star}{\hbar^2}}\sum_{\alpha=0}^{N-1}|\psi_\alpha(y)|^2\sqrt{E_{\mathrm{F}} - E_\alpha} \tag{9}$$

where we have introduced the Fermi energy E_{F}. The factor 2 accounts for spin degeneracy. The Poisson equation for the potential $U(y, z)$ is solved on the (y, z) plane by using finite elements in a rectangular box with Von Neumann boundary conditions on the sides and fixed Dirichlet boundary conditions for the side-gate voltage V_{SG}. The size of the box has been chosen such that the results are free from finite size effects. An example of calculation of the Green's function $G(y, y') \equiv G(y, z = 0; y', z' = 0)$ is shown in Supplementary Fig. 3 for illustration. The Schrödinger equation is discretized using a simple finite difference scheme and solved using the Kwant package[4]. Solving the sequence of Supplementary Eq. (7), (8) and (9) for an input density ρ results to a new density ρ^{out}. The self consistency is reached when $\rho = \rho^{\mathrm{out}}$ (see Supplementary Figure 5 for an example of convergence of our iterative procedure). The self-consistent solutions are obtained using a Newton-Raphson scheme[5]. We use the following parameters: effective mass $m^\star = 0.067\, m_e$, dielectric constant $\epsilon = 12\,\epsilon_0$ and a fixed dopant density of $n_d = 3.16 \times 10^{11}\,\mathrm{cm}^{-2}$. Note that this density is higher than the bulk 2D density of the gas. We have checked that our results are in fact independent of this value since the actual electronic density is controlled by V_{SG}. However, using a lower dopant density prevents us from exploring the high density regime ($V_{\mathrm{SG}} > -1\,\mathrm{V}$) where the

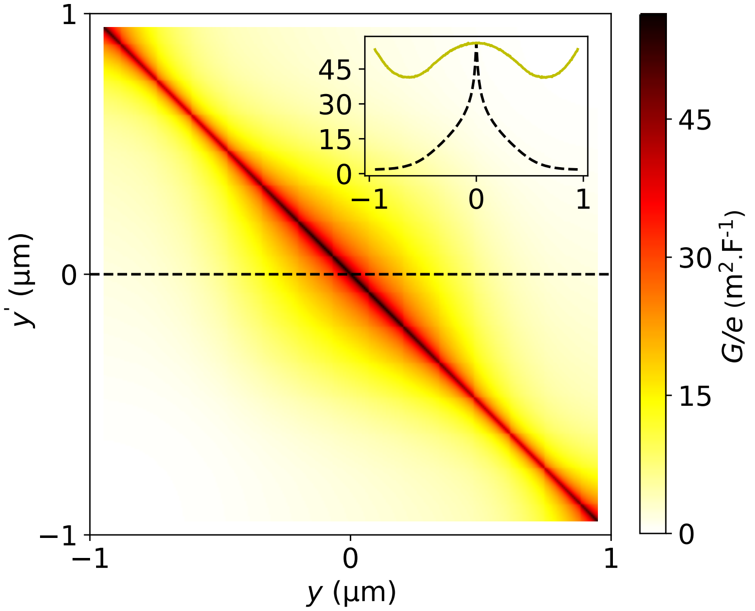

Supplementary Figure 3. **Green's function profile**. The Green's function $G(y, y')$ of the Poisson equation calculated with the finite element method. The two thin regions in the diagonal of the figure at $y, y' = \pm 0.5$ coincide with the positions underneath the electrostatic gate. In the inset, the black dashed line represents a horizontal cut $G(y, y' = 0)$ while the solid yellow line is the diagonal part of the matrix $G(y, y)$.

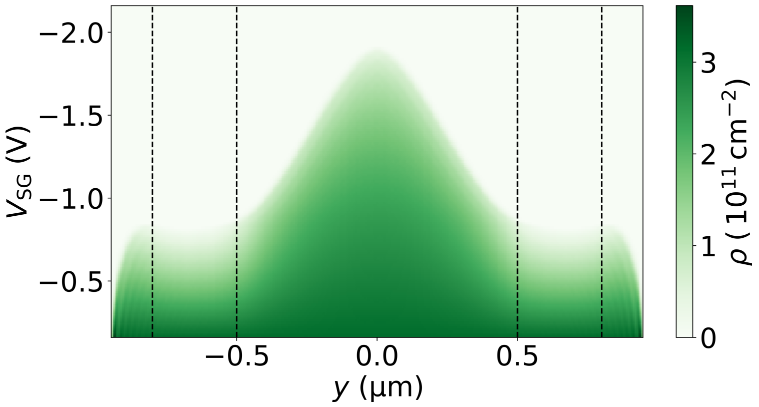

Supplementary Figure 4. **Density profile in the long quasi-1D channel.** Colormap: density as a function of the transverse direction of the wire y (µm) and external gate voltage V_{SG} (V). The vertical black dashed lines show the positions of the two top gates.

quasi-1D wire is not defined anymore. Supplementary Figure 4 shows a color map of the electronic density as a function of the transverse direction of the wire y and V_{SG}. The critical values of the gate voltage where the wire forms ($V_{\mathrm{SG}} \sim -0.8\,\mathrm{V}$, the gas is depleted beneath

the gates) and the pinch-off ($V_{\mathrm{SG}} \sim -1.8\,\mathrm{V}$, full depletion) can be clearly identified.

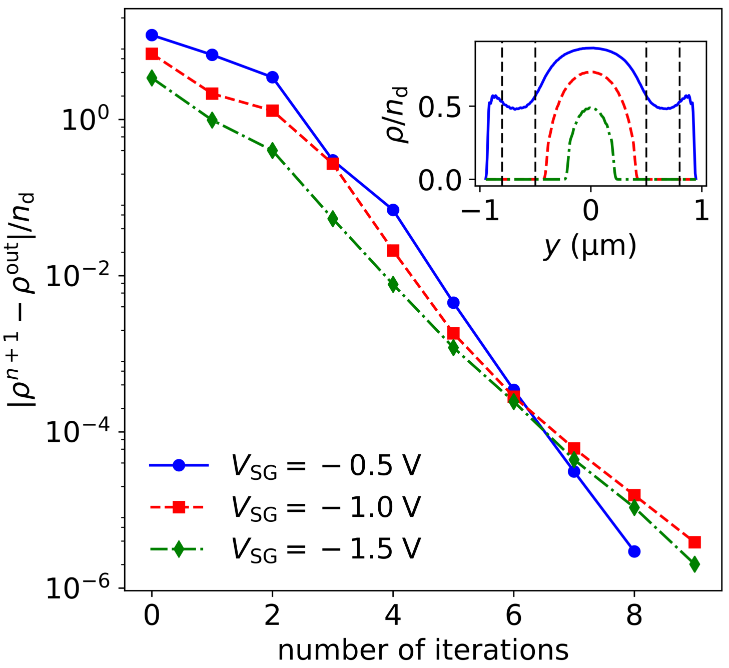

Supplementary Figure 5. **Convergence of our iterative procedure.** Convergence of the self-consistent algorithm for three different gate voltages (solid blue: -0.5 V, dashed red: -1.0 V and dash-dotted green: -1.5 V). At each iteration n we calculate from an input density ρ^n a new density ρ^{n+1}. Solving sequentially Supplementary Equations (7, 8 and 9) we obtain ρ^{out} from which we can calculate the distance to the convergence and express it in terms of the dopant density $n_{\mathrm{d}} = 3.16 \times 10^{11}\,\mathrm{cm}^{-2}$. The inset is the converged density for the 3 gate voltages in the same units.

Generalized Luttinger theory

To proceed, we follow Matveev and Glazman[6] (see also a simpler construction[7]) and construct the bosonised theory for the plasmon excitations of the quasi-1D wire. Bosonization theory predicts that the plasmons have a linear dispersion relation $\omega = v_{\mathrm{P}} q$, where ω is the plasmon energy, v_{P} the plasmon velocity and q the plasmon wave vector. The values of v_{P} are obtained from an eigenvalue problem described below.

In the presence of N propagating channels, we introduce the $N \times N$ diagonal velocity

matrix $\tilde{V}$

$$\tilde{V}_{\alpha\beta} = \delta_{\alpha\beta} v_\alpha \tag{10}$$

where v_α is the non-interacting velocity of mode α. We also introduce the interaction matrix $\tilde{G}$ defined as,

$$\tilde{G}_{\alpha\beta} = \sqrt{v_\alpha v_\beta} \int \mathrm{d}y\mathrm{d}y' \, |\psi_\alpha(y)|^2 G(y, y') |\psi_\beta(y')|^2 \tag{11}$$

Supplementary Figure 6 and 7a show examples of the different components of $\tilde{V}$ and $\tilde{G}$ for different values of the gate voltage. Once these objects have been defined, the plasmon velocities v_P can be obtained in a straightforward manner by diagonalising the following matrix,

$$\left(\tilde{V}^2 + \frac{2}{h}\tilde{G}\right) \tilde{\mathbf{n}} = v_{\mathrm{P}}^2 \tilde{\mathbf{n}} \tag{12}$$

where $\tilde{\mathbf{n}}$ is a N-sized vector. Typically Supplementary Eq. (12) has one large eigenvalue – referred to as the plasmon mode with velocity $v_{\mathrm{P}} = v_{\mathrm{P}}^0$ – and $N-1$ small ones (the slow modes) due to the low effective rank of the $\tilde{G}$ matrix (see Supplementary Fig. 6). The plasmon velocity v_{P} is the chief outcome of this calculation and is shown in Supplementary Fig. 7b. By selecting the $N_{\mathrm{ch}} \times N_{\mathrm{ch}}$ submatrix obtained by truncating Supplementary Eq. (12) to the corresponding channels, we reproduce the effect of the channel filtering with $\mathrm{QPC_0}$. Diagonalising the 1×1, the 2×2 and the full matrix give respectively the red, green and blue curves of Fig. 2b of the manuscript. In the same way, in order to obtain the theory data of Fig. 3d at $V_{\mathrm{SG}} = -1$ V, we solve the $N_{\mathrm{ch}} \times N_{\mathrm{ch}}$ truncated equation where $N_{\mathrm{ch}}(V_{\mathrm{QPC_0}})$ is the number of opened channels of $\mathrm{QPC_0}$.

Modeling of the observed signal

The generalized Luttinger theory provides two pieces of information: the velocities v_P^a of the different modes and the eigenfunctions $\tilde{n}_{a\alpha}$ that indicate how mode a decomposes on the different single particle channels α. A proper theory of how these modes are generated by the ohmic contact, affected by the presence of the intermediate QPC and eventually measured with the last QPC is beyond the scope of this article. Below, we investigate two limiting cases where we predict the actual shape of the measured signal within a (i) "Funneling" scenario and a (ii) "Filtering" scenario. In both scenarios, we assume that the Ohmic contact initially populates all single particle states equally, which amounts to

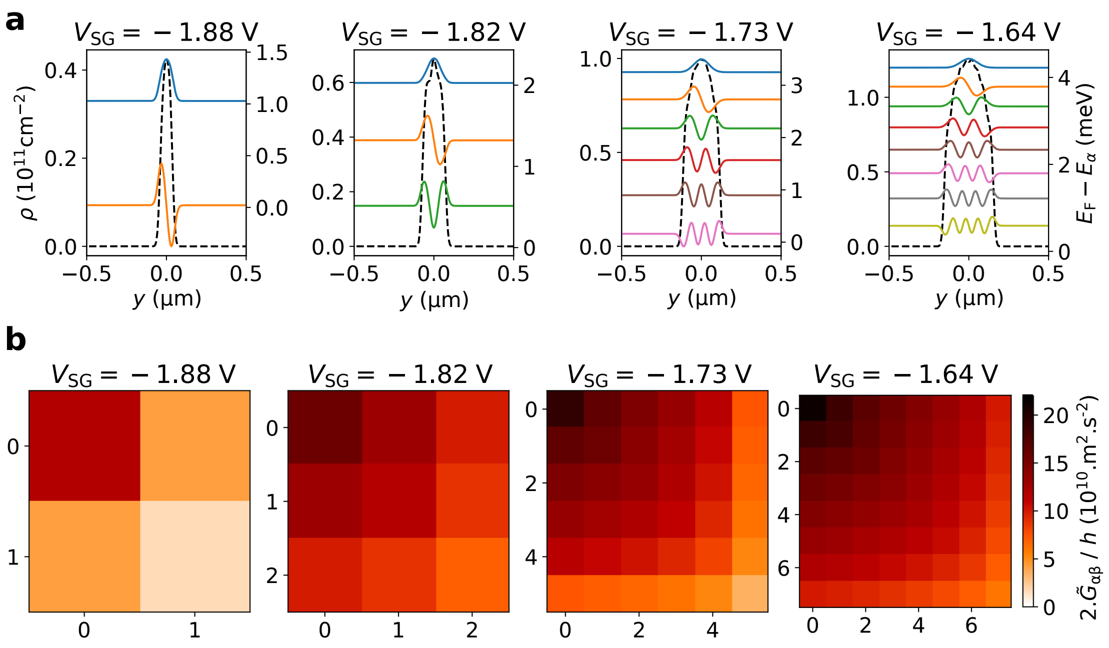

Supplementary Figure 6. **Wave functions in the quasi-1D channel and effective interaction matrix. a**, Density and wave functions for four different channel gate voltages (−1.88, −1.82, −1.73 and −1.64 V). In each plot the black dashed curve represents the self-consistent density ρ in cm^{-2} (left y axis). Each coloured line sketches the shape of the wave function (in arbitrary units) of one open mode ψ_α, centred around its kinetic energy $E_\mathrm{F} - E_\alpha$ (right y axis). **b**, Green function matrix $\tilde{G}$ from which we calculate the renormalized velocities, Supplementary Eq. (12).

assuming that mode a receives an initial weight $c_a = \sum_\alpha \tilde{n}_{a\alpha}$ (where the global sign of $\tilde{n}_{a\alpha}$ is fixed by imposing $c_a \geq 0$).

(i) Funneling scenario: Within this scenario, the charge hosted by the different channels is funneled into the lowest channel upon entering a QPC (a part can also be reflected, here we are not interested in the absolute height of the signal but in the relative weight of the different modes). At a distance d, the expected measured signal takes the form,

$$S(t) \propto \sum_{a=0}^{N-1} c_a f\left(t - \frac{d}{v_\mathrm{P}^a}\right) \quad (13)$$

where $f(t) = \exp(-4\log(2)t^2/\Gamma^2)$ with a FWHM (Full Width Half Maximum) $\Gamma = 68\,\mathrm{ps}$. Here $f(t)$ corresponds to the Gaussian shape of the injected charge pulse (see Fig. 2a and 5b of the manuscript). The corresponding signal is plotted in the upper left panels of Supplementary Figure 8 a, b and c.

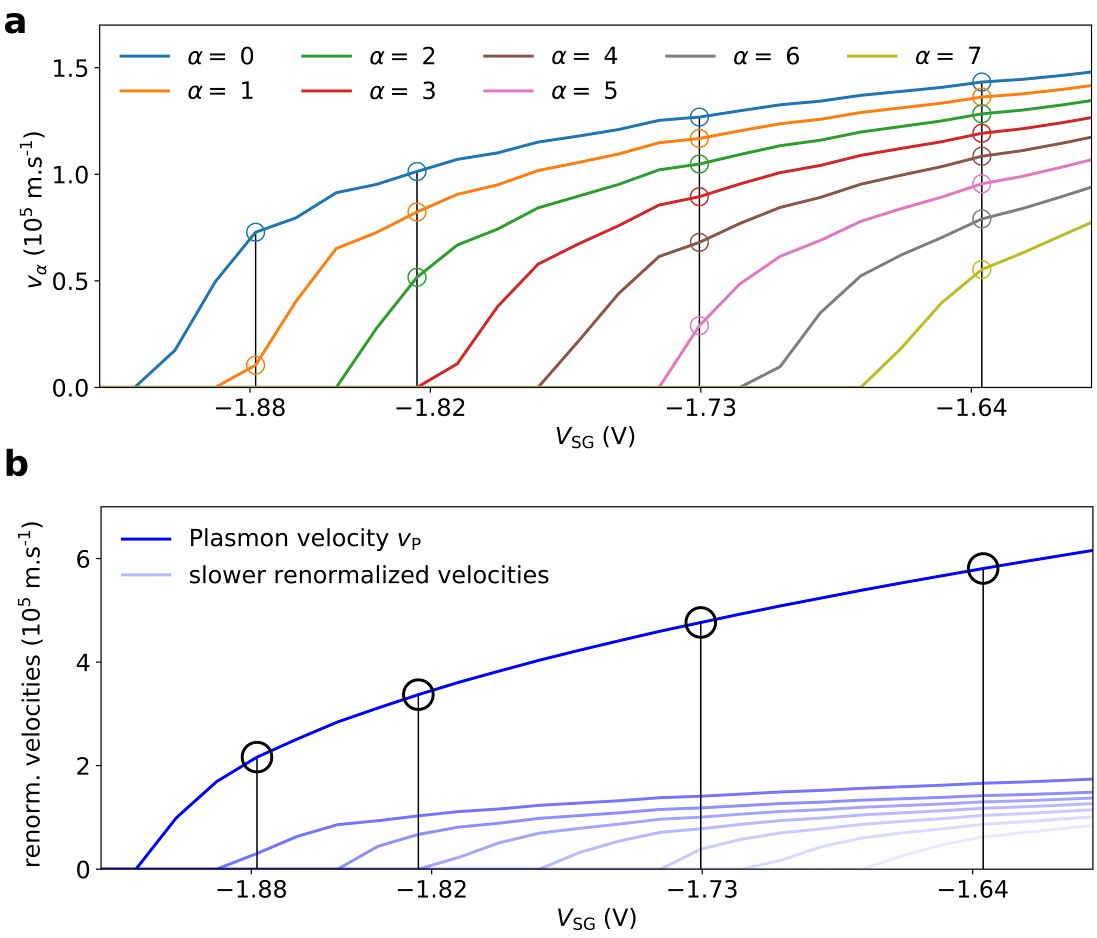

Supplementary Figure 7. **Velocities before and after renormalization.** **a**, Non-interacting velocities v_α for the open channels as a function of gate voltage V_{SG}. The four gate voltages (-1.88, -1.82, -1.73 and $-1.64\,\mathrm{V}$) are marked with black vertical lines and the velocities of the corresponding open modes are marked with circles. **b**, Renormalized velocities v_{P}^a calculated from Supplementary Eq. (12) for each gate voltage V_{SG}. One velocity, indicated by the solid blue line, is considerably higher than the others. This fast collective mode, propagating with velocity v_{P}, corresponds to the plasmon mode shown in Fig. 2b of the manuscript.

Upon polarizing QPC_0 to a unique transmitting channel $T = 1$, one expects that the plasmon is funneled into a plasmon hosted purely by the first channel. In practice this corresponds to truncating Eq.(12) to a 1×1 matrix before diagonalizing the problem. We expect

$$S(t) \propto f\left(t - \frac{d}{v_{\mathrm{P}}^{(1\times1)}}\right) \tag{14}$$

where $v_{\mathrm{P}}^{(1\times1)}$ refers to the truncated matrix. The corresponding signal is plotted in the upper panels of Supplementary Figure 8 a, b and c. The velocity of this pulse corresponds to the red curve shown in Fig. 2b. A similar calculation with a truncation to a 2×2 matrix leads to the green curve of Fig. 2b.

(ii) Filtering scenario: Upon entering a QPC polarised to transmission $T = 1$, only the weight of the mode corresponding to the lowest channel (channel 0) is transmitted, the rest is reflected. This corresponds to adding a factor $\tilde{n}_{a0}$ in the expected signal. The fast plasmon mode has a large charge weight c_0 compared to the other modes. However this charge is (more or less equally) distributed over all the single particle channels. In contrast, the other (slow) modes have spread their weight on a few channels with both positive and negative contributions. As a result, the extra factor $\tilde{n}_{a0}$ strongly reduces the overall weight of the fast mode compared to the slow ones. The expected weight without selection QPC_0 reads,

$$S(t) \propto \sum_{a=0}^{N-1} c_a \tilde{n}_{a0} f\left(t - \frac{d}{v_{\mathrm{P}}^a}\right) \tag{15}$$

while with selection QPC_0 we expect,

$$S(t) \propto \sum_{a=0}^{N-1} c_a (\tilde{n}_{a0})^2 f\left(t - \frac{d}{v_{\mathrm{P}}^a}\right). \tag{16}$$

These signals are plotted respectively on the lower panels of Supplementary Figure 8 a, b and c for different distances.

At a short distance (d$<$ 10 µm) one observes only a single charge pulse when using the selection QPC_0 which is consistent with both scenarios. For a longer distance ($d > 20$ µm), on the other hand, one observes a clear splitting of the signal into two separate peaks of comparable magnitude ("fractionalisation") for the filtering scenario. This is in contrast to what we see experimentally when measuring the charge pulse at QPC_2 which is placed at a distance of d$=$ 25 µm from the selection QPC_0. At this distance we only observe a Gaussian charge pulse with a single peak and which propagates with a much slower speed compared to the case when not using the selection QPC_0. This lets us conclude that the Funneling scenario is consistent with our experimental data. For even longer distances one observes complete separation of the fast plasmon mode and the slow modes. The speed of this fast charge mode can be measured when QPC_0 is depolarized and corresponds to the blue curve of Fig. 2b of the main text.

Note that our conclusion stands if we replace the velocities predicted by our modeling by the one actually measured: the fastest velocity that we measure (larger than $8 \times 10^5\,\mathrm{m\,s^{-1}}$) is sufficiently different from the slow ones (less than $2 \times 10^5\,\mathrm{m\,s^{-1}}$) for two peaks of FWHM $= 68\,\mathrm{ps}$ show a clear splitting after propagating more than $25\,\mathrm{\mu m}$.

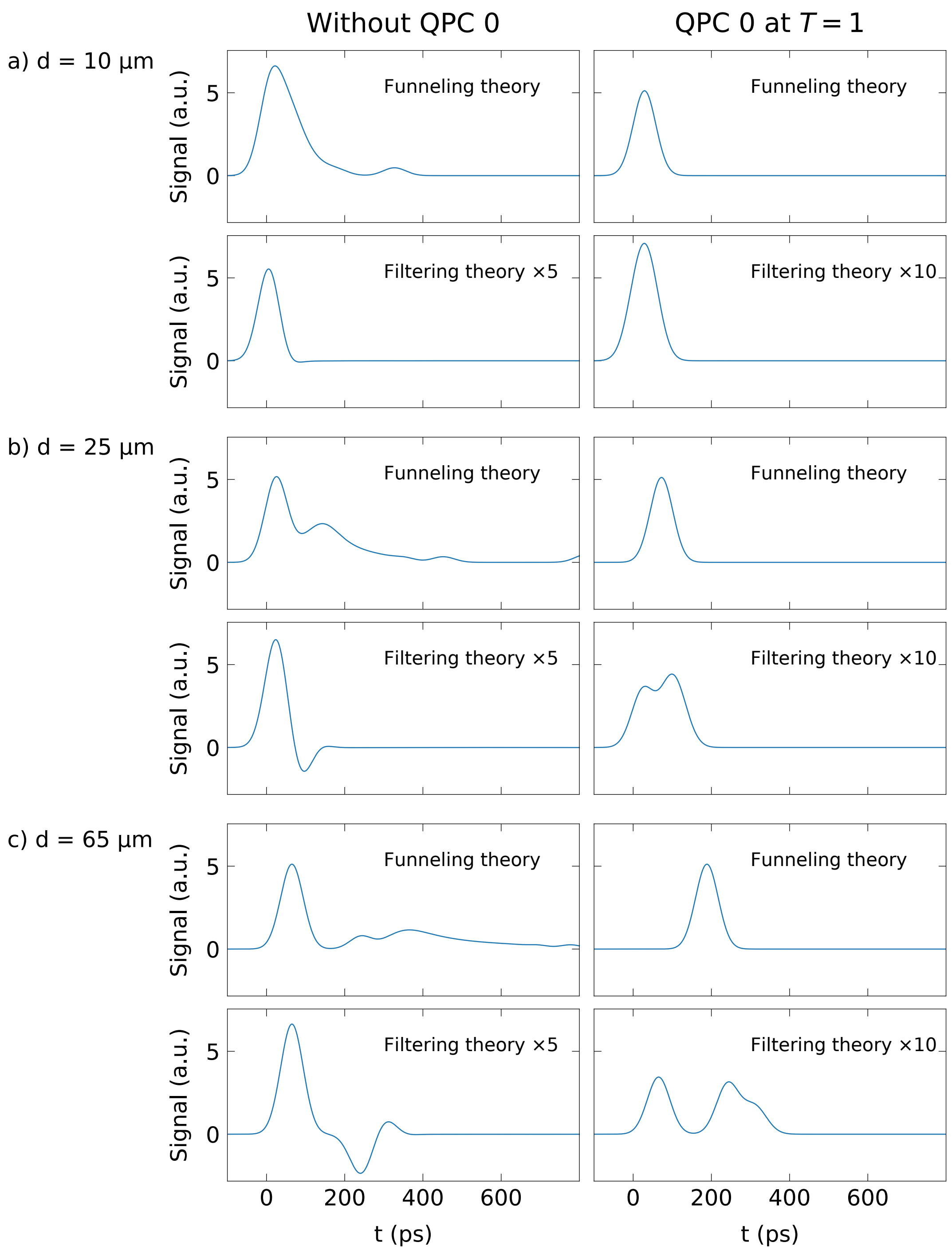

Supplementary Figure 8. **Modeling the observed Signal.** Expected signal for the two scenarios (Funneling and Filtering) and two experimental situations (measurement with or without polarising QPC_0). The curves correspond to $V_{SG} = -1.0\,V$ and a distance of $d = 10\,\mu m$ (a), $d = 25\,\mu m$ (b) and $d = 65\,\mu m$ (c).

REFERENCES

[1]Gaury, B. & Waintal, X. Dynamical control of interference using voltage pulses in the quantum regime. *Nature Communications* **5**, 3844 (2014).

[2]Chaplik, A. V. Absorption and emission of electromagnetic waves by two-dimensional plasmons. *Surface Science Reports* **5**, 289–335 (1985).

[3]Wu, J. *et al.* Excitation, detection, and electrostatic manipulation of terahertz-frequency range plasmons in a two-dimensional electron system. *Scientific reports* **5**, 15420 (2015).

[4]Groth, C. W., Wimmer, M., Akhmerov, A. R. & Waintal, X. Kwant: a software package for quantum transport. *New Journal of Physics* **16**, 063065 (2014).

[5]Ben-Israel, A. A newton-raphson method for the solution of systems of equations. *Journal of Mathematical Analysis and Applications* **15**, 243–252 (1966).

[6]Matveev, K. A. & Glazman, L. I. Conductance and coulomb blockade in a multi-mode quantum wire. *Physica B: Condensed Matter* **189**, 266–274 (1993).

[7]Kloss, T., Weston, J. & Waintal, X. Transient and Sharvin resistances of Luttinger liquids. *Phys. Rev. B* **97**, 165134 (2018).

[8]Freulon, V. *et al.* Hong-Ou-Mandel experiment for temporal investigation of single-electron fractionalization. *Nature Communications* **6**, 6854 (2015).
